# The prevalence of intestinal dysbiosis in patients referred for antireflux surgery

**DOI:** 10.1007/s00464-020-08229-5

**Published:** 2021-01-21

**Authors:** Jordan J. Haworth, Nicholas Boyle, Andres Vales, Anthony R. Hobson

**Affiliations:** 1 The Functional Gut Clinic, London, W1G 6NB UK; 2 RefluxUK, London, UK

**Keywords:** Gastroesophageal reflux disease (GERD), Proton pump inhibitors (PPIs), Small intestinal bacterial overgrowth (SIBO), Bloating

## Abstract

**Background:**

Prior to antireflux surgery, most patients with symptoms of gastroesophageal reflux disease (GERD) have been taking long-term proton pump inhibitors (PPIs). PPIs have been shown to cause changes to the intestinal microbiota, such as small intestinal bacterial overgrowth (SIBO), which is characterised by symptoms of gas bloating. Patients undergoing antireflux surgery are not routinely screened for SIBO, yet many patients experience gas-related symptoms postoperatively.

**Methods:**

Data from consecutive patients (*n* = 104) referred to a speciality reflux centre were retrospectively assessed. Patients underwent a routine diagnostic workup for GERD including history, endoscopy, oesophageal manometry and 24-h pH-impedance monitoring off PPIs. Intestinal dysbiosis was determined by hydrogen and methane breath testing with a hydrogen-positive result indicative of SIBO and a methane-positive result indicative of intestinal methanogen overgrowth (IMO).

**Results:**

60.6% of patients had intestinal dysbiosis (39.4% had SIBO and 35.6% had IMO). Patients with dysbiosis were more likely to report bloating (74.6% vs 48.8%; *P* = 0.01) and belching (60.3% vs 34.1%; *P* = 0.01). The oesophageal acid exposure time and number of reflux episodes were similar between dysbiosis and non-dysbiosis groups, but patients with dysbiosis were more likely to have a positive reflux-symptom association (76.2% vs 31.7%; *P* < 0.001), especially for regurgitation in those with SIBO (*P* = 0.01). Hydrogen gas production was significantly greater in patients with a positive reflux-symptom association for regurgitation (228.8 ppm vs 129.1 ppm, *P* = 0.004) and belching (mean AUC 214.8 ppm vs 135.9 ppm, *P* = 0.02).

**Conclusions:**

The prevalence of intestinal dysbiosis is high in patients with GERD, and these patients are more likely to report gas-related symptoms prior to antireflux surgery. Independently, SIBO may be a contributory factor to refractory reflux symptoms and gas bloating in antireflux surgery candidates.

Indications for antireflux surgery include inadequate symptomatic response to high-dose acid suppression, typically proton pump inhibitors (PPIs), or a desire to cease drug therapy due to quality of life considerations. Most patients undergoing antireflux surgery will have been taking PPIs for many years. Indeed, a recent study in patients randomised to antireflux surgery or high-dose PPI therapy reported that the average duration of PPI use for all patients was 8.4 (range 0.3–35) years [1].

The most common postoperative complaints following antireflux surgery are dysphagia and symptoms related to gas-bloat syndrome, although gas-related symptoms are more frequently reported than heartburn, epigastric pain, and dysphagia at 5 years postoperatively [2–4]. Gas-bloat syndrome is characterised by the inability to belch, postprandial bloating and flatulence. Outcomes of bloating and the inability to belch are also considered to be less acceptable than dysphagia by antireflux surgery candidates [5] and may be a reason to decline surgery during the consenting process. While these symptoms are thought to be caused by a super-competent fundoplication that prevents gastric venting, the mechanism for gas-bloat remains unclear.

A multitude of adverse events have been associated with long-term PPI use despite poor evidence and a lack of randomised trials [6]. These include dementia, chronic kidney disease, pneumonia, bone fracture, hypomagnesaemia, vitamin B12 deficiency, gastric cancer, *Clostridium difficile* infection and changes to the gut microbiome.

There is compelling evidence which shows that PPI therapy alters the composition of gut microflora to one that is populated with oropharyngeal commensals and predisposed to enteric gut infections [7, 8]. The use of PPIs has a more profound change on the gut microbiota when compared to antibiotics or any other drug, with a significant shift of the gut microbiome in PPI users towards the oral microbiome [8]. PPIs reduce the acidity of the stomach, thereby reducing the powerful antimicrobial effect of stomach acid and allowing colonisation of microorganisms in the proximal gut. Several studies have demonstrated that PPI use is associated with small intestinal bacterial overgrowth (SIBO) [9–11].

In SIBO, there is an altered or increased presence of bacteria in the small bowel defined by a bacterial count of > 10^3^ colony forming units per mL [12]. SIBO is manifested by symptoms of diarrhoea, postprandial bloating and flatulence, which are consequent to an increase in gas production by bacterial fermentation in the small bowel. Hydrogen and methane breath testing (HMBT) is a simple, non-invasive and a widely accepted means of detecting SIBO. It utilises the detection of hydrogen (H_2_) and methane (CH_4_) gas, which are specific biomarkers of endogenous bacterial fermentation [13]. Excessive methane production is due to an overgrowth of archaea, as opposed to bacteria. Since archaea may overgrow throughout the intestinal tract, the term "intestinal methanogen overgrowth (IMO)" is appropriate to allow a distinction to be made [14].

The cause of gas-bloat symptoms following antireflux surgery may be related to a disruption of the intestinal microbiota, including SIBO, and PPI use may be the precipitant of this. The aim of this retrospective study was to utilise HMBT for the identification of intestinal dysbiosis in patients with PPI-refractory symptoms of GERD who were being reviewed for antireflux surgery.

## Methods

This retrospective database study included patients referred from secondary care to a speciality reflux centre from March 2017 to September 2019. All patients were evaluated by a surgeon and underwent 24-h oesophageal pH-impedance monitoring with a view to potentially undergoing antireflux surgery. All patients who also completed a lactulose hydrogen and methane breath test were selected and the data reviewed. Exclusion criteria included patients younger than 18 years old, use of antibiotics (< 4 weeks), a history of organic gastrointestinal disease, and previous gastrointestinal surgery. Patients with a hiatal hernia were not excluded. All patients included in this study had provided written informed consent at the time of testing that their data could be used anonymously for research and audit purposes.

### Lactulose HMBT

Patients were provided with preliminary instructions before the test. Antibiotics were avoided for 4 weeks before, probiotics and all forms of laxatives were avoided for 1 week before, and anti-diarrhoeal medications were avoided for 2 days before. On the day prior to testing, patients followed a low fermentable diet which included white bread, white rice, white potatoes without skin, maximum two eggs, baked or grilled chicken and white fish, water, unflavoured black tea or coffee and one tablespoon of butter or margarine. No other foods were permitted. A 12-h fast was performed before the test. On the day of the test, patients were advised to brush teeth, avoid smoking or chewing gum and limit to one glass of water. Physical activity was prohibited during the test to keep carbon dioxide (CO_2_) levels constant.

A single baseline breath sample was collected before ingestion of 10 g of lactulose dissolved in 200 mL of water. Thereafter, a further 9 breath samples were collected every 15 min. Breath samples were collected in exetainer vials (Labco, UK), and concentrations of H_2_, CH_4_ and CO_2_ were measured by gas-chromatography (Agilent, Santa Clara, CA). Intestinal dysbiosis was defined by a positive result for SIBO and/or positive result for IMO. A rise in H_2_ ≥ 10 ppm from baseline occurring within 60 min was indicative of SIBO [15], and a measurement of CH_4_ ≥ 10 ppm in any single breath sample during the test was indicative of IMO [12]. Area under the concentration–time curve (AUC) for H_2_ and CH_4_ was calculated by adding the CO_2_-adjusted levels using the trapezoid method.

### Reflux monitoring

Patients were instructed to discontinue PPI medications for 5 days before the test, H_2_ receptor antagonists for 2 days before the test and liquid alginates 1 day before the test. Patients were required to fast for at least 4 h before the test. High-resolution oesophageal manometry was performed prior to 24-h oesophageal pH-impedance monitoring in order to locate the lower oesophageal sphincter (LES) for accurate placement of the distal pH sensor 5 cm above the LES. A pH-impedance catheter and ZepHr recorder device (Diversatek, Milwaukee, WI) was used to detect acid, non-acid and gaseous reflux events. An abnormal oesophageal acid exposure time (AET) was defined by an AET > 4.0%, which is a borderline abnormal result according to the Lyon Consensus [16]. The patient’s most troublesome symptoms (up to a maximum of 3) were recorded using buttons on the pH recorder for reflux-symptom association, but lower gastrointestinal symptoms (nausea, bloating and abdominal pain) were not assessed for reflux-symptom association. A positive reflux-symptom association was determined by a symptom index of ≥ 50% together with a symptom association probability of ≥ 95%.

### Statistical analysis

Data were collected retrospectively from the patient’s referral letter, history, HMBT report and 24-h pH-impedance data. Continuous variables were expressed as means ± standard deviation, unless otherwise stated. Discrete variables were expressed as numbers and percentages. The means were compared by student’s *t*-tests. The association between characteristics was determined by the Pearson chi-squared tests. A *P* value of < 0.05 was considered statistically significant. All statistical analyses were performed using IBM SPSS Statistics 24.

## Results

104 patients were included in this study. The mean age of the study population was 51 (range 21–78) years, and 51.9% were male. All patients had been taking gastric acid antisecretory medication for at least 6-months prior to investigation and still reported at least one typical symptom of GERD (heartburn, regurgitation and/or chest pain). The demographic information is summarised in Table 1. Around half of the patients reported gas-related symptoms with excessive belching in 50.0% and bloating in 64.4%. In total, 63/104 patients (60.6%) had intestinal dysbiosis as determined by HMBT. The proportion of patients with a H_2_-positive (SIBO) and CH_4_-positive (IMO) breath test was 39.4% and 34.6%, respectively. The prevalence of concomitant SIBO and IMO was 13.4%.

**Table 1:** Demographic and 24-h oesophageal pH-impedance characteristics for patients with a negative HMBT and patients with a positive HMBT for intestinal dysbiosis

	Negative HMBT	Positive HMBT	*P* value*
*N*	41 (39.4)	63 (60.6)	
Male	19 (46.3)	35 (55.6)	0.36
Mean (SD) age in years	53.2 ± 14.3	49.2 ± 14.1	0.16
Symptoms			
Heartburn	24 (58.5)	42 (66.7)	0.40
Regurgitation	22 (53.7)	34 (54.0)	0.98
Chest pain	11 (26.8)	23 (36.5)	0.30
Cough	13 (31.7)	16 (25.4)	0.48
Belching	14 (34.1)	38 (60.3)	0.01
Nausea	9 (22.0)	18 (28.6)	0.45
Bloating	20 (48.8)	47 (74.6)	0.01
Abdominal pain	15 (36.6)	30 (47.6)	0.27
AET > 4.0%	10 (24.4)	23 (36.5)	0.19
Mean (SD) number of reflux events	50.4 ± 35.2	61.5 ± 37.0	0.13
Mean (SD) DeMeester score	13.6 ± 18.5	12.3 ± 17.8	0.72
Positive reflux-symptom association	13 (31.7)	48 (76.2)	< 0.001
Heartburn SAP	5 (12.2)	14 (22.2)	0.20
Regurgitation SAP	6 (14.6)	24 (38.1)	0.01
Chest pain SAP	1 (2.4)	3 (4.8)	0.55
Cough SAP	3 (7.3)	1 (1.6)	0.14
Belching SAP	6 (14.6)	23 (36.5)	0.02

Values are expressed as numbers (percentages) unless stated otherwise

*HMBT* hydrogen and methane breath test, *AET* acid exposure time, *SAP* symptom association probability

*Using Student’s *t* test and χ^2^ test between negative and positive HMBT groups

A total of 33 patients had an abnormal acid exposure time on oesophageal pH-metry (Table 1), and this was not statistically significant between the dysbiosis and non-dysbiosis groups (*P* > 0.05). The mean number of reflux events was slightly higher (61.5 ± 37.0) in patients with dysbiosis than in those without (50.4 ± 35.2), but this was not statistically significant (*P* > 0.05). The mean DeMeester score was not different between dysbiosis and non-dysbiosis groups (*P* > 0.05). A total of 61 patients had a positive reflux-symptom association, and this was statistically significant between dysbiosis and non-dysbiosis groups (*P* < 0.001). Patients with a positive reflux-symptom association had significantly more H_2_ production on HMBT than those without (mean AUC 199.3 ppm vs 99.2 ppm; *P* = 0.001). A positive reflux-symptom association for regurgitation and belching was more frequent in those with a positive HMBT compared to those with a negative HMBT (*P* = 0.01 and *P* = 0.02, respectively). Further analysis determined that breath H_2_ production was greater in patients with a positive reflux-symptom association for regurgitation (mean AUC 228.8 ppm vs 129.1 ppm, *P* = 0.004) and belching (mean AUC 214.8 ppm vs 135.9 ppm; *P* = 0.02). The AUC for CH_4_ was not different between groups (*P* > 0.05). Symptoms and oesophageal pH-metry findings for patients with a H_2_-positive or CH_4_-positive breath test are shown in Table 2.

**Table 2:** Symptoms and pH-impedance characteristics for patients with a H_2_-positive breath test and patients with a CH_4_-positive breath test

	H_2_-positive	*P* value	CH_4_-positive	*P* value*
*N*	41 (39.4)		36 (34.6)	
Symptoms				
Heartburn	27 (65.9)	0.68	25 (69.4)	0.36
Regurgitation	22 (53.7)	0.98	18 (50.0)	0.57
Chest pain	14 (34.1)	0.80	15 (41.7)	0.16
Cough	9 (22.0)	0.28	11 (30.6)	0.66
Belching	23 (56.1)	0.32	24 (66.7)	0.01
Nausea	14 (34.1)	0.13	8 (22.2)	0.53
Bloating	33 (80.5)	0.01	23 (63.9)	0.93
Abdominal pain	21 (51.2)	0.19	15 (41.7)	0.81
AET > 4.0%	13 (31.7)	0.99	13 (36.1)	0.49
Mean (SD) number of reflux episodes	59.1 ± 31.0	0.67	63.2 ± 42.7	0.22
Mean (SD) DeMeester score	11.4 ± 19.6	0.51	11.3 ± 13.0	0.53
Positive reflux-symptom association	32 (78.0)	< 0.001	26 (72.2)	0.04
Heartburn SAP	10 (24.4)	0.19	7 (19.4)	0.82
Regurgitation SAP	18 (43.9)	0.01	11 (30.6)	0.78
Chest pain SAP	0 (0.0)	0.10	3 (8.3)	0.08
Cough SAP	4 (3.8)	0.10	1 (2.8)	0.68
Belching SAP	15 (36.6)	0.11	12 (33.3)	0.37

Values are expressed as numbers (percentages) unless stated otherwise

*H*_*2*_*-positive* hydrogen-positive breath test, *CH*_*4*_*-positive* methane-positive breath test, *AET* acid exposure time, *SAP* symptom association probability

*Using Student’s *t* test and χ^2^ test between H_2_-positive and H_2_-negative groups and CH_4_-positive and CH_4_-negative groups

No statistical differences were seen between dysbiosis and non-dysbiosis groups for the type of reported symptoms, except for gas-related symptoms of belching and bloating. In patients with dysbiosis, 60.3% reported belching compared to 34.1% in those without dysbiosis on HMBT (*P* = 0.01), and 74.6% of patients with dysbiosis reported bloating compared to 48.8% of patients without dysbiosis (*P* = 0.01). Further analysis determined that total gas production on HMBT was greater in patients reporting bloating (298.4 ppm vs 145.9 ppm, *P* = 0.002) and patients reporting belching (299.1 ppm vs 202.5 ppm, *P* = 0.02) compared to patients who did not report bloating or belching, respectively.

Patients were also categorised by the results of their oesophageal pH-metry. A total of 26/35 patients (74.3%) with reflux hypersensitivity (negative AET and positive symptom association) and 23/33 patients (69.7%) with proven GERD (positive AET with or without a positive symptom association) had dysbiosis on HMBT. The prevalence of SIBO in these groups was 57.1% and 39.4%, and the prevalence of IMO was 37.1% and 39.4%, respectively.

## Discussion

Our data suggest that nearly two-thirds of patients with reflux symptoms, who have been taking long-term PPIs, have intestinal dysbiosis that is recognisable on HMBT, be it SIBO, IMO or a combination of both. This adds to the growing body of evidence that long-term PPI use may lead to changes in the gut microbiota [7–11].

To our knowledge, these findings are the first to suggest that refractory GERD symptoms may be consequent to changes in the gut microbiota, and there appears to be an increased likelihood of a positive reflux-symptom association in patients with dysbiosis, especially for regurgitation, which is a common indication for antireflux surgery [17]. The presence of SIBO, i.e. a H_2_-positive breath test, was independently associated with a positive reflux-symptom association for regurgitation. In addition, patients with a positive symptom association for regurgitation and belching had greater production of hydrogen on HMBT. Therefore, we postulate that SIBO may be a contributory factor to refractory reflux symptoms owing to endogenous bacterial fermentation in the small bowel. Methanogens can be found in the small bowel but are thought to predominantly overgrow in the colon [18], which may explain why methane production was not associated with reflux.

Furthermore, at 5 years following fundoplication, the prevalence of gas-bloat syndrome is around 40% [4]. Interestingly, this is similar to the prevalence of SIBO in our study. The cause of gas-bloat syndrome following fundoplication is thought to be secondary to the inability of the gastroesophageal junction to relax in response to gastric distension since gas reflux events are less frequent after a partial fundoplication than after a complete fundoplication [19]. However, the exact pathophysiology of gas bloat is unknown. Our data suggest that gas-related symptoms prior to antireflux surgery may be consequential to SIBO since bloating was independently associated with a H_2_-positive breath test.

H_2_ and CH_4_ are by-products of bacterial metabolism, which are absorbed from the gastrointestinal tract and exhaled in the breath making them exclusive biomarkers of endogenous intestinal bacteria. While a CH_4_-positive breath test is irrefutable due to the requirement of only one single breath sample measuring ≥ 10 ppm, the false-positive rate for a H_2_-positive breath test with glucose or lactulose is high, especially in the presence of rapid mouth-to-cecum transit time [20, 21]. Therefore, we opted to use a more conservative cut-off (a rise of H_2_ within 60 min as opposed to 90 min) to reduce the likelihood of a false-positive SIBO result [15]. The prevalence of SIBO in our study is comparable to that across studies that looked at the association between PPI use and SIBO [22].

HMBT is simple and inexpensive, yet they are not routinely performed in the workup of antireflux surgery patients. Incorporation of breath tests into the workup algorithms of reflux patients under consideration for surgery may be of benefit and guide treatment options. To our knowledge, there is no published data assessing outcomes following SIBO eradication in patients presenting with refractory reflux symptoms either before or after antireflux surgery. However, it is possible that identification and treatment of these patients may avoid the need for surgery or even predict a better side-effect profile following antireflux surgery. Of note, one retrospective study showed approximately 30% of patients undergoing Nissen fundoplication had SIBO, and these patients had inferior quality of life scores, namely gas-related symptoms [23].

Prior to antireflux surgery, patients are required to undergo oesophageal pH testing to provide confirmatory evidence of reflux disease. Consequently, we categorised our patients based on the results of their oesophageal pH-impedance test. The prevalence of SIBO was 57.1% in the reflux hypersensitivity group and 39.4% in the proven GERD group. Reflux hypersensitivity was defined by normal acid exposure but a positive correlation between the patient’s symptoms and reflux events on pH-impedance monitoring [24]. Patients with proven GERD had an abnormal AET with or without a positive symptom association. Although, studies have shown that patients with a positive reflux-symptom association, independent of AET, have favourable outcomes for antireflux surgery [25, 26]. Therefore, this study suggests that around 1 in 2 good candidates for antireflux surgery have SIBO. A positive symptom association for regurgitation would typically bolster the decision for antireflux surgery, but we showed that a positive symptom association for regurgitation may be related to H_2_ production from SIBO. The evaluation of SIBO in patients with postoperative gas-bloat symptoms should be considered as a focus for further study.

While SIBO can be difficult to treat, a recent guideline has identified dietary modification and antibiotics can be effective [14]. In a randomised double-blind placebo-controlled trial, patients with abdominal bloating had a positive response to rifaximin and symptom improvement correlated to a reduction in H_2_-breath excretion [27]. Rifaximin is a non-systematic antibiotic that is superior to other antibiotics for the treatment and retreatment of SIBO [28]. However, one study found that 66% of irritable bowel syndrome (IBS) patients who did not eliminate CH_4_ with rifaximin alone were able to normalise HMBT following the addition of neomycin [29].

Rifaximin is mostly reserved for the treatment of traveller’s diarrhoea, hepatic encephalopathy and IBS-D. However, Tan et al., found that rifaximin led to relief of belching and postprandial fullness/bloating in patients with functional dyspepsia in the absence of IBS [30]. There is a well-established overlap between GERD and IBS patients due to functional dyspepsia symptoms, namely belching and bloating [31, 32]. Our study found that belching and bloating were more common in reflux patients with intestinal dysbiosis, and gas production was significantly greater in these patients on HMBT. Changes to the intestinal microbiome appear to play an important role in the aetiology of IBS [33, 34]. The efficacy of rifaximin in IBS is presumed to be an antibiotic effect on the small intestinal microbiota, especially since it is more efficacious in the presence of bile acids [35]. Hence, the overlap between GERD and IBS may be derived from PPI-induced changes to the proximal intestinal microbiota. This is further supported by Zhong et al., who demonstrated that bacterial load in the duodenum is correlated positively with symptom severity in dyspeptic patients [36].

There were several limitations within this study. First, we did not know the status of intestinal dysbiosis prior to PPI therapy, and some patients had been taking PPIs for over 10 years. Second, there was no healthy control group, albeit there is a lack of conclusive data for the prevalence of intestinal dysbiosis in patients not taking PPIs, particularly when considering both SIBO and IMO. The study by Lombardo et al., found the prevalence of SIBO in 50 healthy controls, who had not taken PPIs for at least 10 years, to be 6.0% on HMBT [9]. Although, this was using glucose, which is considered to be more sensitive for diagnosing SIBO when compared to lactulose [13]. To overcome this, we used a more conservative cut-off time in this study as previously described. Third, there was no outcome data in those who eventually opted for surgical intervention. Hence, we are planning to carry out a prospective study on treating patients with intestinal dysbiosis to see if there is a reduction in reflux symptoms.

Of note, we did not exclude patients with aerophagia, and supragastric belching is thought to be the main determinant of troublesome belching in GERD [37]. However, we only found seven patients who demonstrated pathological supragastric belching and four were in the negative HMBT group [38]. Therefore, the primary cause of belching in reflux patients may be due to fermentative intestinal dysbiosis.

## Conclusion

The prevalence of intestinal dysbiosis is high in patients who are under consideration for antireflux surgery. This study highlights a potentially novel connection between bacterial fermentation patterns and refractory GERD symptoms, namely regurgitation. It adds to the growing body of evidence that gastric acid suppression may alter the intestinal microbiota. Since there appears to be a relationship between SIBO and bloating in antireflux surgery candidates, breath testing could be useful in the pre-surgical workup by determining those who may be more prone to gas-bloat syndrome. These findings warrant further research into the role that changes in the intestinal microbiota, especially untreated SIBO, may have in GERD patients who undergo antireflux surgery. Also, it highlights the need to identify pathways for the treatment of SIBO, either before or after antireflux procedures, or perhaps to help avoid surgery altogether.
